# Measuring facility readiness to provide childbirth care: a comparison of indices using data from a health facility survey in Ethiopia

**DOI:** 10.1136/bmjgh-2021-006698

**Published:** 2021-10-05

**Authors:** Elizabeth K Stierman, Saifuddin Ahmed, Solomon Shiferaw, Linnea A Zimmerman, Andreea A Creanga

**Affiliations:** 1 Department of International Health, Johns Hopkins University Bloomberg School of Public Health, Baltimore, Maryland, USA; 2 Department of Population, Family And Reproductive Health, Johns Hopkins University Bloomberg School of Public Health, Baltimore, Maryland, USA; 3 School of Public Health, Addis Ababa University, Addis Ababa, Oromia, Ethiopia; 4 Department of Gynecology and Obstetrics, Johns Hopkins School of Medicine, Baltimore, Maryland, USA

**Keywords:** health services research, health systems, obstetrics, maternal health, child health

## Abstract

**Background:**

Actionable information about the readiness of health facilities is needed to inform quality improvement efforts in maternity care, but there is no consensus on the best approach to measure readiness. Many countries use the WHO’s Service Availability and Readiness Assessment (SARA) or the Demographic and Health Survey (DHS) Programme’s Service Provision Assessment to measure facility readiness. This study compares measures of childbirth service readiness based on SARA and DHS guidance to an index based on WHO’s quality of maternal and newborn care standards.

**Methods:**

We used cross-sectional data from Performance Monitoring for Action Ethiopia’s 2019 survey of 406 health facilities providing childbirth services. We calculated childbirth service readiness scores using items based on SARA, DHS and WHO standards. For each, we used three aggregation methods for generating indices: simple addition, domain-weighted addition and principal components analysis. We compared central tendency, spread and item variation between the readiness indices; concordance between health facility scores and rankings; and correlations between readiness scores and delivery volume.

**Results:**

Indices showed moderate agreement with one another, and all had a small but significant positive correlation with monthly delivery volume. Ties were more frequent for indices with fewer items. More than two-thirds of items in the relatively shorter SARA and DHS indices were widely (>90%) available in hospitals, and half of the SARA items were widely (>90%) available in health centres/clinics. Items based on the WHO standards showed greater variation and captured unique aspects of readiness (eg, quality improvement processes, actionable information systems) not included in either the SARA or DHS indices.

**Conclusion:**

SARA and DHS indices rely on a small set of widely available items to assess facility readiness to provide childbirth care. Expanded selection of items based on the WHO standards can better differentiate between levels of service readiness.

**Key questions**

**What is already known?**

- Many health facilities in low-income and middle-income countries operate under significant constraints, such as inadequate staffing, medicine stock-outs, equipment shortages and poorly functioning information and referral systems, which limit their capacity to provide safe and effective childbirth care.
- Information about the readiness of health facilities to provide childbirth care is needed to guide quality improvement efforts, but there is no consensus on the best approach to measure readiness.

**What are the new findings?**

- This study compares three facility survey assessment tools and statistical methods for constructing indices to measure facility childbirth service readiness in Ethiopia and finds that indices show moderate agreement with one another.
- More than two-thirds of items in the relatively shorter tools were widely (>90%) available in hospitals in Ethiopia, limiting the ability of the tools to discriminate between readiness levels.
- Items based on the WHO quality of care standards showed greater variation and captured unique aspects of readiness not included in other indices.

**What do the new findings imply?**

- It is feasible to create a service readiness index without the use of complex statistical methods; additive methods produce indices that are easy to generate, interpret and deconstruct to identify bottlenecks to health system performance.
- Item selection should favour inclusion of items with a strong theoretical basis and the ability to discriminate between levels of service readiness.

## Introduction

Building on momentum to end preventable maternal and newborn deaths, country and global stakeholders have committed to meet the Sustainable Development Goals (SDGs) of reducing the global maternal mortality ratio to less than 70 deaths per 100 000 live births and neonatal mortality rates to 12 or fewer deaths per 1000 live births in all countries by 2030.^1^ Achievement of these targets will depend on improving coverage of life-saving interventions during the intrapartum period and the first 24 hours following birth, when an estimated 46% of maternal deaths and 40% of neonatal deaths and stillbirths occur.^2^

Improving skilled birth attendance, primarily through increasing the proportions of births at health facilities, is a key intervention for achieving the SDG-3 goals. A recent analysis of household survey and routine health information system data show an increase in the global proportion of deliveries that occur in a health facility from 65% in 2006–2012 to 76% in 2014–2019, with the largest increases observed in sub-Saharan Africa and South Asia.^3^ However, increased use of facility childbirth services has not consistently translated into the expected gains in maternal and neonatal survival. Research offers mixed evidence of the relationship between use of facility childbirth services and maternal and newborn health outcomes in low-income and middle-income countries (LMICs).^4–12^ For maternal health, these inconsistent findings may, in part, be explained by differences in the risk profile of patients accessing services,^4 5 9 10^ with high-risk patients being more likely to seek care at a health facility, therefore, biasing the results towards the appearance of limited or no effectiveness. The mixed evidence also points to significant variations in the quality of care provided across facilities and contexts. Secondary analysis of two large population-based cluster-randomised control trials in Ghana found no evidence of an association between facility birth and mortality outcomes, but the overall result masked differences in quality of care across facilities; proximity to facilities offering high-quality care was associated with lower risk of intrapartum stillbirth and composite mortality outcomes.^7^ Indeed, the quality of childbirth care is now receiving heightened attention globally.^13^

Ensuring facility readiness is an essential first step towards improving the quality of care in LMICs. Readiness, as conceptualised by WHO, is the capacity of a facility to provide services to a defined minimum standard, including the presence of trained staff, commodities and equipment; appropriate systems to support quality and safety; and provider knowledge.^14^ Kanyangarara *et al*’s analysis of survey data from health facilities in 17 LMICs found wide variation in the availability of such essential resources. For example, the availability of magnesium sulphate—a drug used to prevent or treat seizures for patients with (pre-)eclampsia—ranged from 10% to 97% across countries.^15^ Moreover, inadequate provider knowledge and poor adherence to clinical practice standards exacerbate deficiencies in the provision of quality care.^16–20^ As a result, large gaps exist between ‘service contact’ (ie, individuals who use childbirth services) and ‘effective coverage’ (ie, individuals who experience a positive health gain from using childbirth services) in maternal and newborn health in LMICs.^21–23^

For childbirth services, several indices have been proposed to measure service readiness. The WHO’s health facility assessment tool, Service Availability and Readiness Assessment (SARA), proposes indices to measure basic and comprehensive obstetric care readiness.^24 25^ The Demographic and Health Surveys (DHS), the major source of data on population, health and nutrition in LMICs, have also collected facility level data in selected countries using the Service Provision Assessment (SPA) tool since 1999. The SPA surveys cover facility readiness in terms of infrastructure, resources and management systems for antenatal care, delivery services, newborn care and emergency obstetric care. Wang *et al* offer an alternative obstetric and newborn care readiness index in an analytical study using the DHS data.^26 27^ Others have measured readiness to perform obstetric signal functions based on the framework for monitoring emergency obstetric care developed by the WHO, United Nations Population Fund (UNFPA), UNICEF and the Mailman School of Public Health Averting Maternal Death and Disability programme,^28–33^ expanded by some to include signal functions for routine childbirth and newborn care as well as emergency referrals.^34–40^ More recently, researchers and practitioners have proposed using indicators from the WHO’s Standards for improving quality of maternal and newborn care in health facilities to assess a broader range of quality domains.^41–45^

These measurement approaches share many commonalities. However, there are important differences in item selection and aggregation methods and, to date, there is no consensus on the best approach for measuring facility readiness for childbirth services in LMICs. Conventional indices tend to focus on the availability of commodities with limited consideration of the systems necessary to support quality and safety. These conventional indices may not fully capture the readiness elements predictive of quality care. A previous study by Leslie *et al* found that service readiness, based on an index constructed from SARA tracer items, was weakly associated with observed clinical quality of care in Kenya and Malawi.^46^ The need to refocus health facility assessments to measure quality of care—including key readiness, process and outcomes measures—has been a key consideration in the ongoing process to revise the DHS SPA as well as the process led by the WHO, in collaboration with the Health Data Collaborative, to develop a standardised health facility assessment.

Health authorities require actionable information about the readiness of health facilities to guide quality improvement efforts, but there is no agreement on how best to measure readiness. The objective of this study is to compare childbirth service readiness indices to ascertain their relative utility for programming and decision making.

## Methods

### Study setting

The study is based on data from health facilities in Ethiopia. The public sector health service in Ethiopia is designed as a three-tiered system. In rural areas, the primary level consists of an interconnected network of health posts, health centres and primary hospitals, with linkages to general and specialised hospitals.^47^ In urban areas, health centres are linked directly to general hospitals and specialised hospitals. The public sector provides labour and delivery services at health centres and hospitals. Government health centres provide routine delivery services and basic emergency obstetric and neonatal care (BEmONC); government hospitals provide comprehensive emergency obstetric and neonatal care (CEmONC),^48^ which includes caesarean sections and blood transfusions.^47 48^ However, in practice, gaps exist in the capacity of health facilities to provide the full range of obstetric and neonatal care services. A 2016 survey found that only 5% of government health centres were able to provide all BEmONC signal functions, and only 52% of government hospitals had the capacity to offer all CEmONC components.^48^

The private health sector in Ethiopia encompasses a heterogeneous mix of private-for-profit, non-profit and faith-based hospitals and clinics. However, the 2014 SPA-Plus survey estimated that less than one-third of private-for-profit facilities offer labour and delivery services.^49^ These services are generally limited to routine delivery services; few private facilities have the capacity to provide emergency care.^48 49^ Among women who delivered in a health facility, the Ethiopia Mini DHS 2019 estimated that 95% of women delivered in a public facility and only 5% delivered in a private facility.^50^

### Study design and procedures

The study uses cross-sectional data collected between September and December 2019 from a sample of service delivery points (SDPs) across all regions and two city administrations in Ethiopia. SDPs were identified following selection of the study’s enumeration areas (EAs) as described in the study protocol available elsewhere.^51^ All government health posts, health centres, and primary level and general hospitals whose catchment area covers a sampled EA were eligible for the survey. In addition, private sector SDPs located within the EA’s kebele—the lowest level administrative division in Ethiopia—were invited to participate in the survey, up to a maximum of three private SDPs per EA. Private health facilities are relatively rare in rural Ethiopia, and few women in Ethiopia deliver in private facilities.^50^ Our sample reflects this reality, where most kebeles in the Performance Monitoring for Action Ethiopia (PMA-ET) sample did not have even one private SDP.

After obtaining consent from the head of the facility or designated authority, data were collected using a standardised questionnaire, publicly available at http://www.doi.org/10.34976/kvvr-t814.^52^ A total of 534 hospitals, health centres and health clinics completed the survey, a response rate of 98.9%; among these, 406 facilities provide childbirth services. The survey was administered as part of PMA-ET, a project implemented by the Addis Ababa University School of Public Health and the Johns Hopkins Bloomberg School of Public Health, and funded by the Bill & Melinda Gates Foundation (INV 009466).

### Measurement

Selection of items for the childbirth service readiness indices followed existing guidance or theoretical frameworks (online supplemental table S1). The first approach to item selection relies on tracer items for basic and comprehensive obstetric care listed in the WHO SARA Reference Manual^24^; these items were selected by WHO in consultations with service delivery experts.^25^ A second approach uses items included in the index developed by Wang *et al* for a DHS analytical study^26 27^; item selection for this index was also guided by the WHO SARA Reference Manual,^24^ as well as the recommendations by the Newborn Indicator Technical Working Group and a review conducted by Gabrysch *et al*.^40^

10.1136/bmjgh-2021-006698.supp1 Supplementary data

In the third approach, PMA-ET items were mapped to the WHO Standards for improving quality of maternal and newborn care in health facilities^41 53^ to identify a pool of 67 candidate items for health centres/clinics and 79 candidate items for hospitals. Analyses were performed to identify a smaller, parsimonious set of items that would capture the three ‘provision of care’ standards (evidence-based practices, information systems, referral systems) and two ‘cross-cutting’ standards (human resources, physical resources) in the WHO framework.^41 53^ To assess the value of candidate items, we first calculated the percentages of hospitals and the percentages of health centres/clinics that had each item available at the time of the 2019 PMA survey. We excluded items that were nearly universally (>97%) available since these items had limited ability to differentiate between facilities, and we excluded items flagged for concerns about response bias or with unclear interpretations (online supplemental table S2). After this initial round of exclusions, we examined the correlation structure between items overall and by readiness domain: (1) equipment, supplies and amenities; (2) medicines and health commodities; (3) staffing and systems for quality and safety; and (4) performance of signal functions. For each domain, a two-parameter logistic item response model was fitted to characterise item discrimination (ie, the ability of the item to differentiate between facilities of different readiness levels) and item difficulty (ie, whether the item is widely or rarely available in facilities irrespective of readiness level). The final set of 44 items for health centres and 52 items for hospitals was determined based on statistical properties and conceptual alignment with the WHO framework (online supplemental tables S1, S2). Retained items showed variation across facilities and good discrimination, and together, the selection ensured representation across the four readiness domains and five WHO standards.

A scoping review of published and grey literature identified three common approaches for aggregating items to generate a single composite readiness score for childbirth care: simple addition of items, domain-weighted addition of items and the data dimensionality reduction method of principal components analysis (PCA). We paired each of the three-item selection methods with the three aggregation methods to generate nine indices (table 1). Prior to aggregation, all items were coded as 0 ‘no’ or 1 ‘yes’ to indicate whether the item was observed on the day of the assessment, whether the function was reported as performed, or whether the system was reported as being in place. The few instances (<1%) where a response was missing or where interviewees responded ‘don’t know’ were coded to 0. Additionally, five items were only asked for a subset of health facilities (eg, government facilities) and marked ‘not applicable’ for the remainder (3%–8%). For those facilities, the ‘not applicable’ items were excluded and the denominator adjusted accordingly to calculate scores using simple or weighted addition; the ‘not applicable’ responses were coded to 0 prior to aggregation by PCA.

**Table 1:** Methods to construct service readiness indices

**Item selection**	
SARA tracer items for obstetric care*	15 items for health centres plus seven additional items for hospitals based on the WHO SARA basic and comprehensive obstetric care tracer items.^23^ These correspond to three readiness domains: (A) staff and training; (B) equipment and (C) medicines and commodities.
DHS analytical study’s obstetric and newborn care readiness indicators*	30 items for health centres plus three additional items for hospitals based on obstetric and newborn readiness indicators described in the DHS analytical studies No. 65.^24 25^ This includes items across five readiness domains: (A) performance of signal functions for emergency obstetric care; (B) performance of newborn care functions; (C) general requirements; (D) equipment and (E) medicines and commodities. The DHS programme proposes an additional domain for ‘guidelines, staff training and supervision’; however, this domain is excluded from the domain-weighted addition given limited availability of these items in the PMA-ET survey.
WHO standards for improving quality of maternal and newborn care readiness items*	44 items for health centres plus eight additional items for hospitals available in the PMA-ET survey instrument mapped to the WHO Standards for improving quality of maternal and newborn care in health facilities.^39^ These include three ‘provision of care’ standards and two ‘cross-cutting’ standards: (1) evidence-based practices for routine care and management of complications; (2) actionable information systems; (3) functional referral systems; (4) competent, motivated human resources and (5) essential physical resources. These items are also grouped in four readiness domains: (A) equipment, supplies and amenities; (B) medicines and health commodities; (C) staffing and systems for quality and safety; and (D) performance of signal functions.
**Aggregation method**	
Simple addition of items	The number of items that is available on the day of the assessment is added together. The number of available items is divided by the total number of possible items to compute a score ranging from 0 to 1. Each item is given equal weight.
Weighted addition of items by readiness domain	Within each readiness domain, the number of items that is available on the day of the assessment is added together. The number of available items per domain is divided by the number of possible items per domain to compute a domain score. The sum of the domain scores is divided by the number of domains to compute a score ranging from 0 to 1. Each domain is given equal weight.
Principal components analysis (PCA)	PCA is a data reduction technique that converts a set of correlated items into orthogonal components. Each component explains some proportion of the variation across the items, with the first component explaining the largest proportion. The first component is extracted and rescaled to a score ranging between 0 and 1.
**Composite indices**	
Combination of item selections with aggregation methods	Each of the item selections (1=SARA, 2=DHS, 3=WHO standards) are aggregated using three different methods (1=simple addition, 2=weighted addition, 3=PCA) to generate nine childbirth service readiness indices.

*Please refer to online supplemental tables S1 and S6 for the complete list of items selected for each of the readiness indices and for information on any items excluded due to lack of available data.

DHS, Demographic and Health Survey; PMA-ET, Performance Monitoring for Action Ethiopia; SARA, Service Availability and Readiness Assessment.

Readiness scores were calculated separately for hospitals and for health centres/clinics to reflect the difference in services provided at different levels of Ethiopia’s tiered health system. In addition to routine childbirth services, health centres offer BEmONC whereas hospitals offer CEmONC that includes caesarean sections and blood transfusions.^47^ Thus, readiness scores for hospitals were computed using an expanded list of items relevant for CEmONC. Similarly, PCA scores were generated separately for hospitals and for health centres/clinics. As a result, scores are comparable within each tier, but not directly comparable across tiers.

### Statistical analysis

We calculated readiness scores for each health facility in the sample using all nine indices. We then compared measures of central tendency, spread, skewness and kurtosis across approaches. We also examined eigenvalues and loadings for indices generated using PCA in order to assess the variance explained by the first component, subsequently used to calculate the readiness score.

To compare the variability and distribution of scores across indices, we adopted an approach similar to that used by Sheffel *et al* to develop quality of antenatal care indices. 54 Ideally, an index can accurately differentiate between facilities with differing levels of readiness, including those at the high and low ends. To assess this characteristic, we calculated the coefficient of variation and proportion of facilities scoring either a 0 (floor) or 1 (ceiling). Another desirable characteristic is that the individual items that comprise an index demonstrate a range of variability. We assess this by calculating the proportion of items that are rare (<40%) or widely available (>90%).

We calculated differences between readiness scores and between rankings within health facilities measured using different indices and compared these differences using graphical displays. We expected facilities to consistently score high or low regardless of the methods used to assess their readiness. If an index score deviates substantially relative to other indices, this likely indicates that it is measuring a different construct or that particular item(s) are unduly influencing the score. Next, to understand differences in the data structure and composition of the indices, we deconstructed the composite scores into domain-specific scores, and then we examined interdomain correlations, interitem correlations, and the internal consistency of items overall and within each domain.

Prior research suggests an association between childbirth service readiness and delivery volume.^26^ We evaluated this association using Spearman’s ranked correlation coefficient. All statistical analyses were performed using Stata IC, V.15.1.^55^

### Patient and public involvement

Patients were not involved in the research. A project advisory board, chaired by the Deputy Minister of Health, and composed of representatives from the Federal Ministry of Health, professional associations, multilateral organisations, non-governmental organisations and donors provided input during survey design and development. The project advisory board advises PMA-Ethiopia on data analysis, utilisation and dissemination.

## Results

Of the 406 facilities that provide childbirth services, the vast majority are public facilities: 96.3% of hospitals and 93.9% of health centres and clinics (table 2). Facilities are distributed across all regions of the country, with a higher proportion located in the more populous regions of Oromiya, Amhara and the Southern Nations, Nationalities and Peoples Region (SNNP).

**Table 2:** Sample characteristics

	Hospitals (n=160)		Health centres/ clinics (n=246)	
	n	%	n	%
**Managing authority**				
Government	154	96.3	231	93.9
Private	6	3.8	15	6.1
**Teaching status**				
Teaching facility	23	14.4	n/a	n/a
**Region**				
Addis	5	3.1	24	9.8
Afar	6	3.8	10	4.1
Amhara	33	20.6	51	20.7
Benishangul-Gumuz	3	1.9	9	3.7
Dire Dawa	3	1.9	12	4.9
Gambella	4	2.5	7	2.8
Harari	3	1.9	5	2.0
Oromiya	38	23.8	51	20.7
SNNP*	38	23.8	43	17.5
Somali	5	3.1	6	2.4
Tigray	22	13.8	28	11.4

*Includes facilities located in the newly formed Sidama region. The survey was administered in 2019 prior to the ratification of regional statehood for Sidama; data reflects the regional distribution at the time of data collection.

n/a, not applicable; SNNP, Southern Nations, Nationalities, and Peoples Region.

**Figure 1:**
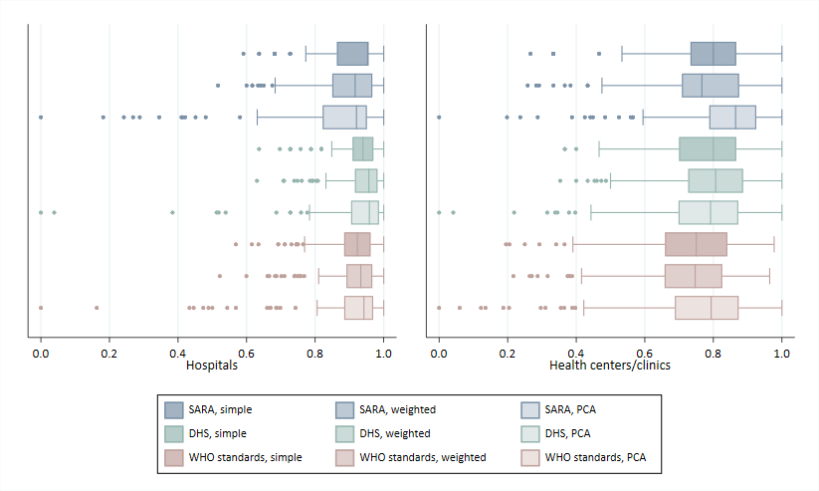
Comparison of childbirth service readiness index scores. DHS, Demographic and Health Survey; PCA, principal components analysis; SARA, Service Availability and Readiness Assessment.

Figure 1 shows the distribution of scores by index (see also online supplemental table 3). Median index scores range from 0.92 to 0.96 for hospitals and from 0.75 to 0.86 for health centres/clinics. WHO standards-based indices generate slightly lower median scores relative to other indices. Across indices, scores show substantial skewness and kurtosis, with observations clustered around the highest scores (online supplemental figure S1). Scores generated using PCA show the greatest skewness and kurtosis.

Scores generated using SARA tracer items show limited item variation and more ceiling effects (table 3); using the SARA simple addition method, 34 (21.2%) hospitals receive a perfect score and 50 (31.2%) tie for the next highest score. Among health centres, 16 (6.5%) receive a perfect score and 45 (18.3%) tie for the next highest rank (online supplemental table S4). The inclusion of more items in the WHO standards-based indices reduces ceiling effects and limits ties in rankings. Use of PCA produces a higher coefficient of variation relative to the simple or domain-weighted addition methods for SARA, DHS and WHO standards-based indices. PCA-derived scores are calculated using the first component, and the eigenvalues for the first component range from 2.5 to 6.6 across indices, explaining 12%–17% of the total variance among items (online supplemental table S5).

**Table 3:** Key characteristics of childbirth service readiness indices

	No of items	Items <40%	Items ≥90%	Coefficient of variation	Floor effects (score=0)	Ceiling effects (score=1)	Correlation with delivery volume	
	n	%	%		%	%	r*	P value
**Hospitals (n=160)**								
SARA tracer, simple	22	0	73	0.10	0	21	0.33	<0.001
SARA tracer, weighted	“	“	“	0.11	0	21	0.32	<0.001
SARA tracer, PCA	“	“	“	0.20	1	<1	0.33	<0.001
DHS, simple	33	0	85	0.07	0	14	0.25	0.002
DHS, weighted†	“	“	“	0.07	0	20	0.20	0.013
DHS, PCA	“	“	“	0.15	1	<1	0.31	<0.001
WHO standards, simple	52	0	67	0.09	0	6	0.23	0.003
WHO standards, weighted	“	“	“	0.09	0	6	0.26	<0.001
WHO standards, PCA	“	“	“	0.16	1	<1	0.29	<0.001
**Health centres/clinics (n=246**)								
SARA tracer, simple	15	7	53	0.17	0	7	0.19	0.004
SARA tracer, weighted	“	“	“	0.19	0	7	0.18	0.004
SARA tracer, PCA	“	“	“	0.18	1	7	0.20	0.002
DHS, simple	30	3	33	0.16	0	1	0.35	<0.001
DHS, weighted†	“	“	“	0.16	0	2	0.39	<0.001
DHS, PCA	“	“	“	0.20	1	1	0.36	<0.001
WHO standards, simple	44	2	23	0.20	0	0	0.31	<0.001
WHO standards, weighted	“	“	“	0.20	0	0	0.35	<0.001
WHO standards, PCA	“	“	“	0.24	1	<1	0.31	<0.001

*Spearman’s correlation coefficients.

†Weighted addition of DHS items excluded the domain for ‘guidelines, staff training and supervision’ given limited information on these items for this sample.

DHS, Demographic and Health Survey; PCA, principal components analysis; SARA, Service Availability and Readiness Assessment.

Individual items contribute different levels of information to the index. Items that are almost universally available—such as fetal scopes, sharps containers, sterile gloves, delivery beds and toilets—provide little information to differentiate between health facilities (online supplemental table S6). Over 70% of items that comprise the SARA and DHS indices are widely available (>90%) in hospitals, and half of items that comprise the SARA index are widely available (>90%) in health centres/clinics (table 3). A slight but significant positive correlation is observed between service readiness scores and monthly delivery volume (table 3).

**Figure 2:**
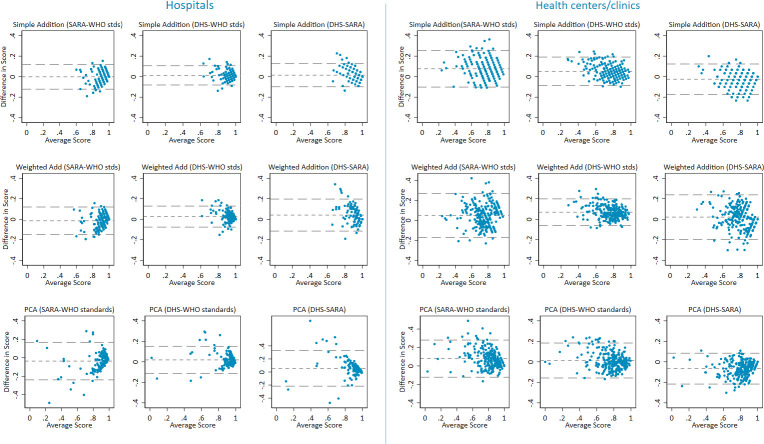
Difference against mean childbirth services readiness Scores. Note: short dashed line indicates mean difference in Readiness Scores and long dashed line indicates 2 SD of the mean difference. DHS, Demographic and Health Survey; PCA, principal components analysis; SARA, Service Availability and Readiness Assessment.

Figure 2 is Bland-Altman graphs that show agreement between service readiness scores generated using different indices (see also online supplemental table S7 and figure S2). There are minimal systemic differences in readiness scores, although WHO standards-based indices produce slightly lower scores than SARA-based or DHS-based indices. SD of differences range from 0.05 to 0.14 among hospitals and from 0.07 to 0.11 among health centres/clinics. DHS and WHO standards-based indices show the greatest consistency in scores, with smaller SD of differences. Among aggregation methods, simple addition produces smaller SD and fewer outliers than PCA and domain-weighted addition.

By and large, there are minimal systemic differences in facility rankings across indices; facilities ranked in the top and bottom tiers by one index are generally ranked similarly by other indices (online supplemental tables S4 and S7). However, some variations do exist, with SARA and WHO standards-based indices displaying the greatest differences in facility rankings (online supplemental figure S2, S3). Additionally, ties are frequent for indices with fewer items, such as the SARA-based indices and, to a lesser extent, the DHS-based indices.

Indices can be deconstructed to measure readiness by their component domains. The SARA and DHS-based indices rely on relatively few items to calculate each domain score and interitem correlation is low; as a result, the internal consistency among items that comprise each domain is weak (online supplemental table S8). Internal consistency improves with the addition of items in the WHO standards-based indices. Across indices, domain-specific rankings generally show slight to moderate correlation with one another (online supplemental table S9). As expected, correlations in domain-specific rankings are highest for domains comprised of similar items (eg, SARA equipment and supplies domain is highly correlated with the WHO standards’ equipment and supplies domain). Meanwhile, the WHO standards’ domain of staffing and systems to support quality, a highly unique domain, exhibits significant but slight correlation with most other domains (r: 0.07–0.30 for hospitals; 0.15–0.43 for health centres). Of note, the DHS newborn signal functions domain appears misaligned to other domains, displaying either no significant or small correlation with other domains.

## Discussion

Our study compares childbirth service readiness scores generated using three different item selection approaches (SARA tracer items, DHS items and WHO standards items) and three-item aggregation methods for each. To our knowledge, it is the first study to compare existing methods for assessing facility readiness using the SARA and DHS guidance and a new method based on the WHO quality of care standards. We find moderate agreement between indices generated using different combinations of items and aggregation methods. Different indices usually produce similar readiness scores—the majority of within-facility scores differ by less than 0.1 on a 0–1 scale—but exceptions occur where scores for the same facility differ by more than 0.4. Importantly, indices also differ in their ability to discriminate between facilities with similar readiness. The SARA-based and DHS-based indices generate more frequent ties and are more prone to ceiling effects, particularly among hospitals with higher levels of readiness. As expected, indices generated using the larger set of WHO standards items produce fewer ties and slightly lower median index scores, a result of selecting items with greater variation across facilities. Among aggregation methods, PCA tends to produce scores with the greatest skewness and kurtosis, and its results are the most difficult to interpret. Other studies have, likewise, found challenges in the use and interpretation of PCA-derived quality of care indices.^54 56^

Differences across the indices arise mainly from differences in item selection and, to a lesser extent, aggregation methods. The DHS and WHO standards-based indices show the greatest agreement. Unlike SARA, these indices include items to measure the past performance of signal functions. We expect service readiness to be closely tied to the ability to perform signal functions when required. A 2016 national assessment of emergency obstetric and newborn care in Ethiopia found that lack of medicines, supplies, equipment and staff were common reasons given by facility staff for not performing a signal function, but other reasons, such as a supportive policy environment and training, were also important.^48^ Past performance of a signal function can be a proxy indicator for these unmeasured elements of readiness and may better predict readiness than the availability of inputs alone, since past performance requires that staff have a minimum level of capacity to recognise and manage obstetric or neonatal emergencies.

Another important difference in the composition of indices is whether they include systems to support quality and patient safety. These include functional referral systems, actionable information systems and processes for continuous quality improvement as conceptualised in the WHO framework for the provision of quality maternal and newborn care.^53^ With the exception of one item related to emergency transport, these systems are not captured in SARA-based or DHS-based indices. Their inclusion in the WHO standards-based indices provides unique information not otherwise captured.

Other differences between indices relate to which medicines are included and how their availability is determined. There are few medicines in the SARA tracer items and these are widely available (eg, oxytocin, magnesium sulfate), whereas WHO standards items include a broader set of medicines for the mother and newborn (eg, BCG vaccine, chlorhexidine gel, dexamethasone/betamethasone, benzathine benzylpenicillin). Of note, SARA and WHO standards-based indices require that medicines be observed in the facility on the day of the assessment, while DHS-based indices require that the medicine be observed in the delivery room.

A key consideration when weighing the merits of a facility readiness index is its usefulness to decision-makers. A good index should provide a clear and accurate overview of readiness, which can be easily deconstructed into its components to assist decision-makers in pinpointing areas of weakness. The SARA-based and DHS-based indices generate domain-specific scores using relatively few items with weak internal consistency; this raises concerns about the robustness of domain-specific scores. Conversely, the greater number items for all readiness domains in the WHO standards-based indices improves internal consistency and generates confidence that domain-specific scores are not excessively sensitive to differences in a single item.

Our study has some limitations. First, health facility assessments are not standardised, and survey items vary across the SARA, SPA and PMA-ET instruments. The PMA-ET survey did not collect data on a few items collected by SARA and SPA (online supplemental table S1). As a result, we are unable to construct the SARA-based and DHS-based indices according to the full list of items referenced in their guidance. Likewise, as recognised in reviews by Brizuela *et al* and Sheffel *et al*,^42 43^ conventional health facility assessments do not generate data to fully measure all standards in the WHO framework; this finding also applies to the PMA-ET survey. As a result, we are unable to consider all potential items that that could be relevant for constructing a WHO standards-based index. In particular, the lack of measures to assess provider knowledge and competency (standard 7 in the WHO framework) is missing across most conventional health facility assessments. While some assessments ask about the receipt of training or supervision, these are not direct measures of provider knowledge or competency. Provider knowledge and competency are, therefore, missing from all facility readiness indices we compared. Second, limited information is available to validate the individual items that comprise the indices. While the majority of items are based on the enumerator’s observation of at least one valid dose or one functional item on the day of assessment per recommended practice,^57^ other items are based on self-reported information prone to recall and other response bias. Third, this study analyses data from a sample of health facilities in one country; results may not be generalisable across other LMIC settings. Finally, traditional epidemiological methods for validating measures are not appropriate for this study—no gold standard exists and the lack of information on individual risk factors complicates assessment against patient outcomes. Instead of validating the index against a traditional gold standard, we considered the face validity and construct validity of indices. Indicative of face validity, items selected for the indices are closely aligned with existing guidelines and the WHO framework for the provision of quality maternal and newborn care, the latter having been developed through an extensive literature review and expert consultations.^41 53^ Indicative of construct validity, service readiness scores are positively correlated with delivery volume.

Our findings have implications for the measurement of service readiness. First, it is feasible to create a service readiness index without the use of complex statistical methods. Simple addition and domain-weighted addition performed better than PCA. These methods produce indices that are easy to generate, interpret and deconstruct to identify bottlenecks to health system performance. Second, indices generated using relatively few items are prone to frequent ties and ceiling effects, a deficiency that is more pronounced when a large proportion of items are almost universally available. The addition of items improves index performance, but should be balanced against the additional data collection burden. Item selection should favour inclusion of high value items with a strong theoretical basis and the ability to discriminate between levels of service readiness. Moreover, we recognise that the availability of medicines, equipment, staff and systems are necessary but not sufficient for the provision of quality care. Incorporating measures of provider knowledge and competency into standard health facility assessment tools—potentially through clinical vignettes as done with the World Bank’s Service Delivery Indicator surveys^58^ or through the observation of real or simulated cases—could better assist decision-makers in identifying and addressing readiness gaps. Understanding the relationship between service readiness, processes of care and outcomes is critical for improving quality and addressing gaps to effective coverage of care during childbirth. Future research by PMA-ET aims to explore these relationships, by linking data on facility readiness to data collected from peripartum women residing in facilities’ catchment area.

## Data Availability

Data are publicly available at https://www.pmadata.org/data/request-access-datasets.
