# Supplementary material for: Measuring facility readiness to provide childbirth care: a comparison of indices using data from a health facility survey in Ethiopia
Source: BMJ Glob Health. 2021 Oct 5;6(10):e006698. doi: 10.1136/bmjgh-2021-006698 (PMC8493923; doi:10.1136/bmjgh-2021-006698)
Supplement: Supplementary data [file bmjgh-2021-006698supp001.pdf]

Table S1: Detailed description of item selection for readiness indices

Index	Items included	Items not included (not available in dataset)
WHO SARA obstetric care tracer items (1)	**Health centers (15 items; 3 domains):**<br>Medicines and commodities (observed in facility): Antibiotic eye ointment (tetracycline) for newborn; Oxytocin; Injectable antibiotic; Magnesium sulphate; Intravenous solution and infusion set;<br>Equipment: Access to a functional ambulance/car on-site for emergency transportation; Sterile cord ties/clamp and scissors/blades (either in sealed delivery kit or separate); Functional suction apparatus for use with catheter and/or manual suction device for fluid extraction; Obstetric forceps and/or functioning electrical vacuum extractor; D&C kit and/or MVA; Self-inflating bag and newborn masks (size 0 and size 1) for resuscitation; Delivery bed; Sterile gloves;<br>Staff and training: FMOH Management protocol on selected obstetric topics, 2010 (2)[1]; Skilled birth attendant present or on call 24h;<br><br>**Hospitals (22 items; 3 domains):** 14 items listed above; and<br>Medicines and commodities: Blood transfusion available at all times facility open (sufficiency); Access to blood bank (safety); Lignocaine/Lidocaine;<br>Equipment: Functional incubator; Sterilization equipment;<br>Staff and training: Skilled provider who can perform C-section present or on call 24h; Skilled provider who can administer anesthesia	**Health centers (4 items):**<br>Examination light; Partograph, Skin disinfectant; Sterilization equipment[2]<br><br>**Hospitals (13[1] items):**<br>Examination light; Partograph, Skin disinfectant; Staff trained in CEmOC; Anesthesia equipment; Blood typing; Cross match testing; Epinephrine; Halothane; Atropine; Thiopental; Suxamethonium bromide; Ketamine
DHS obstetric and newborn care readiness indicators (3)	**Health centers (30 items; 5 domains[3]):**<br>Medicines and commodities (observed in delivery room or nurse/staff station): Injectable antibiotic; Oxytocin; Magnesium sulphate; Intravenous solution and infusion set; Chlorohexidine gel; Antibiotic eye ointment (tetracycline) for newborn;<br>Equipment: Delivery bed; Sterile cord ties/clamp and scissors/blades (either in sealed delivery kit or separate); Functional suction apparatus for use with catheter and/or manual suction device for fluid extraction; Obstetric forceps and/or functioning electrical vacuum extractor; D&C kit and/or MVA; Sterile gloves; Self-inflating bag and newborn masks (size 0 and size 1) for resuscitation; Infant scale; Blood pressure apparatus; Alcohol hand scrub and/or soap and running water for staff handwashing;<br>General requirements: Electricity available at all times when facility was open in last 7 days and/or back-up energy source; Water outlet on site; Functional toilet available for patient use; Skilled birth attendant present or on call 24h; Access to a functional ambulance/car on-site for emergency transportation;<br>Comprehensive emergency obstetric care: Provided parenteral antibiotics for infections related to pregnancy, abortion, labor or delivery in past 3 months; Provided parenteral or oral uterotonics to prevent or treat PPH in past 3 months; Provided parenteral anticonvulsants to	**Health centers (17 items):**<br>Performed removal of retained products in past 3 months; Practices drying and wrapping newborns; Examination light; Partograph; Hydrocortisone; Skin disinfectant; Guidelines for standard precautions; Training in neonatal resuscitation; Training in early and exclusive breastfeeding; Training in newborn infection management; Training in thermal care; Training in cord care; Training in IMPAC; Training in routine care during labor and delivery; Training in CEmOC; Training in AMTSL; Training in

	manage high blood pressure in pregnancy in past 3 months; Performed manual removal of placenta in past 3 months; Provided instrument/ assisted deliveries in past 3 months; Newborn signal functions and immediate care: Performed neonatal resuscitation in past 3 months; Practices skin-to-skin care; Assists mother to breastfeed; Guidelines, staff training, and supervision:[3] FMOH Management protocol on selected obstetric topics, 2010 (2)[4]<br><br>**Hospitals (33 items; domains[3]):** 30 items listed above; and<br>Equipment: Sterilization equipment;<br>Comprehensive emergency obstetric care: Performed blood transfusions for maternity care in past 3 months; Performed cesarean delivery in past 3 months	Kangaroo Mother Care; Supervision<br><br>**Hospitals (17 items):**<br>All above
WHO standards for improving quality of maternal and newborn care readiness items	**Health centers (44 items; 4 domains):**<br>Equipment, supplies, and amenities: Sterile cord ties/clamp and scissors/blades (either in sealed delivery kit or separate); Alcohol hand scrub and/or soap and running water for staff handwashing; Infant scale; Obstetric forceps and/or functioning electrical vacuum extractor; D&C kit and/or MVA; Functional suction apparatus for use with catheter and/or manual suction device for fluid extraction; Self-inflating bag and newborn masks (size 0 and size 1) for resuscitation; Resuscitation table/trolley with light source; Electricity available at all times when facility was open in last 7 days and/or back-up energy source; Private delivery room or visual privacy ensured in delivery area; Newborn corner;<br>Medicines and commodities (observed in facility): Chlorohexidine gel; Injectable vitamin K; Antibiotic eye ointment (tetracycline) for newborn; BCG vaccine; Magnesium sulphate; Calcium gluconate; At least one antihypertensive (hydralazine, nifedipine, or methyldopa); Injectable diazepam, Injectable oxytocin; Misoprostol; Injectable ergometrine; Injectable ampicillin; Injectable gentamicin; Injectable metronidazole; Dexamethasone / betamethasone; Nevirapine (NVP); Benzathine benzylpenicillin;<br>Staffing and systems to support quality: Functional mechanism for reporting data on maternal deaths to the Maternal and Perinatal Death Surveillance Response; Conducts maternal death reviews; Produces monthly reports for the zonal, district, regional, or national HMIS and receives feedback on the facility's HMIS reports that includes recommendations for action; Skilled birth attendant present or on call 24h; Ratio of skilled health personnel to delivery volume meets or exceeds desired levels in FIGO statement (4); Has a performance monitoring team that meets at least quarterly; Access to a functional ambulance/car on-site for emergency transportation; Printed referral form observed; Functional system for recording and sharing outcomes of cases referred in and out;<br>Performance of signal functions: Provided parenteral anticonvulsants to manage high blood pressure in pregnancy in past 3 months; Provided parenteral or oral uterotonics to prevent or treat PPH in past 3 months; Performed manual removal of placenta in past 3 months; Provided instrument/ assisted deliveries in past 3 months; Provided parenteral antibiotics for infections related to pregnancy, abortion, labor or delivery in past 3 months; Performed neonatal	**Health centers and hospitals:**<br>Items:<br>Performed removal of retained products in past 3 months<br><br>Quality statements:<br>2.1 Standardized medical record;<br>3.1 Assessment on admission for referral decision;<br>7.2 Staff competence and skills mix<br><br>Quality standards:<br>"Experience of care" standards are excluded: Effective communication; Respect and preservation of dignity; Emotional support

Stierman EK, *et al. BMJ Global Health* 2021; 6:e006698. doi: 10.1136/bmjgh-2021-006698

	resuscitation in past 3 months; Provided antenatal corticosteroids for fetal lung maturation in past 3 months;<br><br>**Hospitals (52 items; 4 domains):** 44 items listed above; and<br>Equipment, supplies, and amenities: Sterilization equipment; Functional incubator; Oxygen supply; Pulse oximeter;<br><br>Staffing and systems to support quality: Skilled provider who can administer anesthesia;<br>Performance of signal functions: Performed blood transfusions for maternity care in past 3 months; Performed cesarean delivery in past 3 months; Neonatal intensive care provided at facility.	

[1] WHO SARA includes two sets of guidelines: integrated management of pregnancy and childbirth (IMPAC) and comprehensive emergency obstetric care (CEmOC). The PMA Ethiopia survey measures availability of one set of national guidelines that covers both topics: FMOH management protocols on selected obstetric topics (2010).
[2] The PMA-ET survey assessed the availability of sterilization equipment for hospitals but not for health centers.
[3] The DHS program proposes a domain for 'guidelines, staff training, and supervision'; however, only a single item in this domain is available in the PMA-ET survey: FMOH management protocols on selected obstetric topics (2010). This item is included in indices generated using simple addition of items and PCA. The domain is excluded from the index generated using domain-weighted addition given the limited availability of relevant items to calculate a domain score.
[4] DHS includes three sets of guidelines: IMPAC, CEmOC, and preterm labor. The PMA Ethiopia survey measures availability of one set of national guidelines that covers all topics: FMOH management protocols on selected obstetric topics (2010).

Table S2. Selection of items for WHO standards childbirth readiness index

Items	Items included	Notes on items excluded
**Medicines & Commodities**		
Routine Delivery		
Urine dipsticks		Low discrimination
Routine Newborn Care		
Chlorohexidine gel in facility	X	
Injectable vitamin K in facility	X	
Tetracycline ointment in facility	X	
BCG vaccine	X	
Oral polio vaccine		Limited variation (widely available); low discrimination
Basic EmONC		
Magnesium sulphate in facility	X	
Calcium gluconate in facility	X	
At least one antihypertensive (hydralazine, nifedipine, or methyldopa)	X	
Injectable diazepam in facility	X	
Oxytocin in facility	X	
Misoprostol in facility	X	
Injectable ergometrine/methergine in facility	X	
Injectable ampicillin	X	
Injectable gentamicin	X	
Injectable metronidazole	X	
IV solution in facility and infusion set (cannula, needle, and syringe)		Limited variation (widely available); low discrimination
Comprehensive EmONC		
Blood transfusion available at all times facility open		Highly correlated with performance of blood transfusion in past 3 months
Access to blood bank either inside or outside facility		Highly correlated with performance of blood transfusion in past 3 months
Lignocaine/lidocaine		Limited variation (widely available)
Small and Sick Newborn Care		
Dexamethasone / betamethasone	X	
Nevirapine (NVP)	X	
Maternal ARV regimen[4]		Highly correlated with NVP
HIV rapid test		Limited variation (widely available); low discrimination
Benzathine benzylpenicillin (for prevention of congenital syphilis)	X	
Syphilis testing (VDRL)		Low discrimination
**Equipment, Supplies & Amenities**		
Routine Delivery		
Thermometer		Limited variation (widely available); low discrimination
Blood pressure apparatus		Limited variation (widely available); low discrimination
Fetal stethoscope and/or fetal scope		Limited variation (widely available)
Alcohol hand scrub and/or soap and running water for staff handwashing	X	
Sterile gloves		Limited variation (widely available)

Items	Items included	Notes on items excluded
Waste receptable with lid and plastic liner		Low discrimination
Sharps container		Limited variation (widely available)
Already mixed decontaminating solution (0.5% chlorine)		Limited variation (widely available)
Routine Newborn Care		
Sterile cord ties/clamp and scissors/blades (either in sealed delivery kit or separate)	X	
Infant weight scale	X	
Basic EmONC		
Obstetric forceps and/or functioning electrical vacuum extractor	X	
Dilatation and curettage (D&C) kit and/or manual vacuum aspiration (MVA) and cannula	X	
Functional suction apparatus for use with catheter and/or manual suction device for fluid extraction	X	
Self-inflating bag and newborn masks (size 0 and size 1) for resuscitation	X	
Resuscitation table/trolley with light source	X	
Comprehensive EmONC		
Functional sterilizing equipment	X	
Small and Sick Newborn Care		
Functional incubator	X	
Oxygen supply	X	
Pulse oximeter	X	
Amenities – Routine Delivery		
Delivery bed		Limited variation (widely available)
Private delivery room or visual privacy ensured in delivery area	X	
Newborn corner	X	
Water outlet on site		Limited variation (widely available); low discrimination
Electricity available at all times when facility was open in last 7 days and/or back-up energy source	X	
Functional toilet available for patient use		Limited variation (widely available)
**Staffing & Systems to Support Quality**		
Routine Delivery Care		
Skilled birth attendant present at facility or on call 24 hours	X	
Ratio of skilled health personnel to delivery volume meets or exceeds desired scenario in FIGO statement	X	
Management protocols on selected obstetric topics (FMOH, 2010)		Relatively rare; difficult to interpret
Routine Newborn Care		
Baby-friendly guidelines		Relatively rare; difficult to interpret
Comprehensive EmONC		

Items	Items included	Notes on items excluded
Skilled provider available 24h to provide C-section		Highly correlated with performance of c-section in past 3 months; highly correlated with FIGO ratio
At least one staff trained in anesthesia	X	
Small and Sick Newborn Care		
National Comprehensive and Integrated Prevention of Mother-to-Child Transmission of HIV Guideline (FMOH, 2015)		Relatively rare; difficult to interpret
Information Systems & Quality Improvement Processes		
Functional mechanism for reporting data on maternal deaths to the Maternal and Perinatal Death Surveillance Response	X	
Conducts maternal death reviews	X	
Produces monthly reports for the HMIS and receives feedback that includes recommendations for action	X	
Has a performance monitoring team that meets at least quarterly	X	
Referral Systems		
Access to a functional ambulance/car on-site for emergency transportation	X	
Access to phone or radio system at all times		Limited variation (widely available); low discrimination
Printed referral form observed	X	
Functional system for recording and sharing outcomes of cases referred in and out	X	
**Performance of Signal Functions**		
Routine Delivery		
Used partographs to monitor labor		Limited variation (widely available); response bias concerns
Routine Newborn Care		
Skin-to-skin care		Limited variation (widely available); response bias concerns
Assist mother to breastfeed		Limited variation (widely available); response bias concerns
Basic EmONC		
Provided parenteral anticonvulsants to manage high blood pressure in pregnancy	X	
Provided parenteral or oral uterotonics to prevent or treat PPH	X	
Performed manual removal of placenta	X	
Provided instrument/ assisted deliveries	X	
Provided parenteral antibiotics for infections related to pregnancy, abortion, labor or delivery	X	
Performed neonatal resuscitation	X	
Comprehensive EmONC		
Performed blood transfusions for maternity care	X	
Performed cesarean deliveries	X	
Small and Sick Newborn Care		

Stierman EK, *et al. BMJ Global Health* 2021; 6:e006698. doi: 10.1136/bmjgh-2021-006698

**Items**	**Items included**	**Notes on items excluded**
Provided antenatal corticosteroids for fetal lung maturation	X	
Neonatal intensive care provided at facility	X	

Note: Variation refers to how widely available the item is across facilities; low variation indicates that either the item is widely available across facilities (i.e., nearly all have the item) or the item is rarely available across facilities (i.e., nearly all do not have the item) irrespective of readiness level. Discrimination refers to the ability of the item to differentiate between facilities of different levels of readiness.

Table S3: Comparison of childbirth service readiness scores

Item selection	Item aggregation	Hospitals (n=160)			Health centers / clinics (n=246)		
		Median [IQR]	Skewness	Kurtosis	Median [IQR]	Skewness	Kurtosis
SARA tracer items	Simple addition of items	0.95 [0.86-0.95]	-1.32	4.34	0.80 [0.73-0.87]	-1.06	4.59
SARA tracer items	Weighted addition of items	0.92 [0.85-0.97]	-1.35	4.90	0.77 [0.71-0.88]	-0.65	4.02
SARA tracer items	PCA of items	0.92 [0.82-0.95]	-2.56	9.98	0.86 [0.79-0.93]	-2.06	9.32
DHS items	Simple addition of items	0.94 [0.91-0.97]	-1.55	5.77	0.80 [0.70-0.87]	-0.79	3.63
DHS items[1]	Weighted addition of items	0.96 [0.92-0.98]	-1.93	7.39	0.81 [0.73-0.89]	-0.86	3.65
DHS items	PCA of items	0.96 [0.90-0.99]	-4.24	24.89	0.79 [0.70-0.87]	-1.60	7.28
WHO standards items	Simple addition of items	0.92 [0.88-0.96]	-1.82	6.91	0.75 [0.66-0.84]	-0.99	4.03
WHO standards items	Weighted addition of items	0.93 [0.89-0.97]	-2.05	7.99	0.75 [0.66-0.83]	-0.96	3.89
WHO standards items	PCA of items	0.94 [0.89-0.97]	-3.36	16.50	0.79 [0.69-0.87]	-1.51	5.84

[1] Weighted addition of DHS items exclude the domain for 'guidelines, staff training, and supervision' given limited information on these items for this sample.

Stierman EK, *et al. BMJ Global Health* 2021; 6:e006698. doi: 10.1136/bmjgh-2021-006698

Table S4a: Top 25 ranked hospitals by index

	SARA tracer, simple[1]	SARA tracer, weighted[2]	SARA tracer, PCA[3]	DHS, simple[4]	DHS, weighted[5]	DHS, PCA	WHO standards, simple[6]	WHO standards, weighted[7]	WHO standards, PCA
1 (highest)	1	1	3	1	1	5	1	1	2
2	1	1	3	1	1	5	1	1	2
3	1	1	3	1	1	5	1	1	2
4	1	1	3	1	1	5	1	1	2
5	1	1	3	1	1	5	1	1	2
6	1	1	3	1	1	5	1	1	2
7	1	1	3	1	1	5	1	1	2
8	1	1	3	1	1	5	11	16	1
9	1	1	3	1	1	5	11	16	18
10	1	1	3	1	1	5	25	11	11
11	1	1	3	1	1	5	25	11	28
12	1	1	3	1	1	5	28	16	40
13	1	1	3	1	1	5	49	32	24
14	1	1	3	1	1	5	11	26	70
15	1	1	3	1	1	5	54	49	61
16	1	1	3	1	1	5	75	49	45
17	35	66	38	23	1	27	1	1	2
18	35	66	38	23	1	27	1	1	2
19	35	35	70	1	1	5	11	16	29
20	1	1	3	23	108	2	29	29	23
21	35	66	38	23	1	27	11	11	21
22	35	66	38	23	1	27	11	16	18
23	35	48	97	1	1	5	11	16	36
24	35	35	70	1	1	5	29	32	49
25	1	1	3	23	108	2	49	47	31

[1] 34 hospitals are tied for the highest rank using simple addition of SARA tracer items. 50 hospitals are tied for the next highest rank (#35).
[2] 34 hospitals are tied for the highest rank using weighted addition of SARA tracer items. 13 hospitals are tied for the next highest rank (#35).
[3] 34 hospitals are tied for the 3rd highest rank using PCA of SARA tracer items (#3).
[4] 22 hospitals are tied for the highest rank using simple addition of DHS items. 34 hospitals are tied for the next highest rank (#23).
[5] 32 hospitals are tied for the highest rank using weighted addition of DHS items. 10 hospitals are tied for the next highest rank (#33).
[6] 10 hospitals are tied for the highest rank using simple addition of WHO standards items. 14 hospitals are tied for the next highest rank (#11).
[7] 10 hospitals are tied for the highest rank using weighted addition of WHO standards items. 5 hospitals are tied for the next highest rank (#11).

Table S4b: Bottom 25 ranked hospitals by index

	SARA tracer, simple	SARA tracer, weighted	SARA tracer, PCA	DHS, simple	DHS, weighted	DHS, PCA	WHO standards, simple	WHO standards, weighted	WHO standards, PCA
136	129	136	141	123	117	131	111	100	135
137	144	145	128	151	153	127	94	104	79
138	129	136	139	137	149	117	94	126	113
139	144	147	144	123	123	135	114	130	115
140	129	136	141	123	117	131	138	140	138
141	129	118	143	123	138	147	129	136	133
142	129	131	136	154	157	140	129	120	111
143	115	122	109	146	144	153	141	141	143
144	115	118	78	154	150	156	153	152	149
145	129	130	137	137	120	141	151	149	145
146	115	99	110	154	158	158	149	151	152
147	144	147	148	137	134	137	129	134	144
148	148	143	134	146	140	128	146	143	134
149	129	136	113	146	151	139	151	150	150
150	129	143	149	137	144	146	147	147	146
151	153	153	153	151	152	90	159	158	148
152	158	157	155	137	134	142	144	146	147
153	148	156	156	137	137	148	147	148	154
154	153	159	158	137	133	154	150	153	156
155	148	151	151	153	154	151	154	154	153
156	160	160	160	146	144	149	154	156	155
157	153	154	152	154	156	157	156	155	158
158	153	155	154	154	155	155	156	157	157
159	153	151	157	160	160	160	158	159	159
160 (lowest)	158	157	159	159	159	159	160	160	160

Table S4c: Top 25 ranked health centers/clinics by index

	SARA tracer, simple[1]	SARA tracer, weighted[2]	SARA tracer, PCA[3]	DHS, simple[4]	DHS, weighted[5]	DHS, PCA[6]	WHO standards, simple	WHO standards, weighted	WHO standards, PCA
1 (highest)	1	1	1	1	1	1	2	4	2
2	1	1	1	4	10	7	1	1	1
3	1	1	1	1	1	1	5	13	10
4	1	1	1	4	10	4	13	11	9
5	1	1	1	4	10	11	13	7	26
6	17	67	22	4	1	6	2	2	3
7	1	1	1	12	23	16	23	30	20
8	1	1	1	12	32	26	13	32	11
9	17	17	45	4	5	8	13	21	16
10	1	1	1	1	1	1	43	29	77
11	17	17	17	12	18	25	13	6	32
12	17	17	45	4	5	8	23	25	27
13	17	67	22	12	21	12	5	10	7
14	17	67	22	12	7	15	13	15	13
15	17	17	38	12	23	17	23	26	19
16	17	67	22	12	10	19	12	8	25
17	17	17	45	12	36	24	13	23	18
18	1	1	1	4	7	5	68	64	60
19	1	1	1	12	58	14	32	57	36
20	17	17	17	27	43	30	23	27	23
21	17	67	22	27	23	35	13	19	14
22	17	67	22	27	23	34	13	19	17
23	17	67	22	12	10	13	32	37	33
24	1	1	1	12	23	21	68	49	75
25	17	17	38	4	10	10	43	51	71

[1] 16 health centers/clinics are tied for the highest rank using simple addition of SARA tracer items. 45 health centers/clinics are tied for the next highest rank (#17).
[2] 16 health centers/clinics are tied for the highest rank using weighted addition of SARA tracer items. 23 health centers/clinics are tied for the next highest rank (#17).
[3] 16 health centers/clinics are tied for the highest rank using PCA of SARA tracer items. 5 health centers/clinics are tied for the next highest rank (#17).
[4] 3 health centers/clinics are tied for the highest rank using simple addition of DHS items. 8 health centers/clinics are tied for the next highest rank (#4).
[5] 4 health centers/clinics are tied for the highest rank using weighted addition of DHS items. 2 health centers/clinics are tied for the next highest rank (#5).
[6] 3 health centers/clinics are tied for the highest rank using PCA of DHS items. 1 health centers/clinics holds the next highest rank (#4).

Table S4d: Bottom 25 ranked health centers/clinics by index

	SARA tracer, simple	SARA tracer, weighted	SARA tracer, PCA	DHS, simple[1]	DHS, weighted	DHS, PCA	WHO standards, simple	WHO standards, weighted	WHO standards, PCA
222	217	226	217	228	221	227	202	209	193
223	217	236	230	184	170	210	230	235	235
224	217	226	228	200	196	217	222	226	220
225	234	239	236	213	211	222	202	211	203
226	217	221	212	213	215	206	230	228	231
227	206	216	203	228	230	229	222	227	222
228	217	221	220	213	211	218	227	231	234
229	206	216	221	222	228	226	227	234	233
230	217	236	224	239	238	233	215	208	214
231	217	226	233	231	238	234	222	223	223
232	217	226	228	231	234	231	230	229	226
233	234	232	234	222	224	228	237	232	230
234	234	241	241	231	235	240	222	230	215
235	217	226	227	231	240	230	240	240	239
236	217	221	235	239	230	243	237	237	232
237	234	232	232	241	241	237	230	236	225
238	234	232	237	241	243	235	230	233	228
239	234	239	238	231	232	236	230	238	236
240	243	243	243	231	227	239	237	217	238
241	234	232	240	231	232	242	243	244	243
242	234	220	239	241	245	241	242	241	242
243	242	242	242	231	235	238	241	242	241
244	243	245	245	244	244	244	244	245	244
245	246	244	246	245	242	245	245	243	245
246 (lowest)	243	246	244	245	246	246	246	246	246

[1] 2 health centers/clinics are tied for the lowest rank using simple addition of DHS items (#245).

Table S5: Eigenvalues for PCA method

	Eigenvalue for Component 1	Proportion of variance explained
**Hospitals**		
SARA tracer items	3.20	0.16
DHS items	3.73	0.13
WHO standards items	6.03	0.12
**Health centers/clinics**		
SARA tracer items	2.51	0.17
DHS items	3.64	0.12
WHO standards items	6.55	0.15

Stierman EK, *et al. BMJ Global Health* 2021; 6:e006698. doi: 10.1136/bmjgh-2021-006698

Table S6. Summary of item availability in hospitals and health centers/clinics

	Hospital N=160		Health center/ clinic N=246		SARA	DHS	WHO standards
	No.	%	No.	%			
**Medicines & Commodities**							
Routine Newborn Care							
Chlorohexidine gel in delivery room or nurse/staff station	90/159[1]	56.6	119/244[1]	48.8		X	
Chlorohexidine gel in facility	98/159[1]	61.6	133	54.1			X
Injectable vitamin K in facility	151	94.4	183	74.4			X
Tetracycline ointment in delivery room or nurse/staff station	145	90.6	214	87.0		X	
Tetracycline ointment in facility	152	95.0	229	93.1	X		X
BCG vaccine[2]	136	85.0	219	89.0			X
Basic EmONC							
Magnesium sulphate in delivery room or nurse/staff station	149	93.1	173/245[1]	70.6		X	
Magnesium sulphate in facility	153	95.6	181	73.6	X		X
Calcium gluconate in facility	140	87.5	112	45.5			X
At least one antihypertensive (hydralazine, nifedipine, or methyldopa)	158	98.8	210	85.4			X
Injectable diazepam in facility	153	95.6	159	64.6			X
Oxytocin in delivery room or nurse/staff station	152	95.0	209	85.0		X	
Oxytocin in facility	159	99.4	231	93.9	X		X
Misoprostol in facility	144	90.0	123	50.0			X
Injectable ergometrine/methergine in facility	139	86.9	132	53.7			X
Injectable ampicillin	153	95.6	183	74.4			X
Injectable gentamicin	146	91.3	211	85.8			X
Injectable metronidazole	149	93.1	130	52.8			X
At least one broad-spectrum injectable antibiotic: ampicillin or gentamicin	157	98.1	231	93.9	X	X	
IV solution in delivery room or nurse/staff station and infusion set (cannula, needle, and syringe)	149	93.1	211	85.8		X	
IV solution in facility and infusion set (cannula, needle, and syringe)	152	95.0	221	89.8	X		
Comprehensive EmONC							
Blood transfusion available at all times facility open	122	76.3	--	--	X		
Access to blood bank either inside or outside facility	121	75.6	--	--	X		
Lignocaine/lidocaine	156	97.5	--	--	X		
Small and Sick Newborn Care							
Dexamethasone / betamethasone	153	95.6	146	59.3			X
Nevirapine (NVP)	152	95.0	172	69.9			X
Benzathine benzylpenicillin	113	70.6	147/244[1]	60.2			X
**Equipment, Supplies & Amenities**							
Routine Delivery							
Blood pressure apparatus	152	95.0	216	87.8		X	

	Hospital N=160		Health center/ clinic N=246		SARA	DHS	WHO standards
	No.	%	No.	%			
Alcohol hand scrub and/or soap and running water for staff handwashing	149	93.1	223	90.7		X	X
Sterile gloves	156	97.5	243	98.8	X	X	
Routine Newborn Care							
Sterile cord ties/clamp and scissors/blades (either in sealed delivery kit or separate)	160	100	241	98.0	X	X	X
Infant weight scale	156	97.5	235/245[1]	95.9		X	X
Basic EmONC							
Obstetric forceps and/or functioning electrical vacuum extractor	155	96.9	118	48.0	X	X	X
Dilatation and curettage (D&C) kit and/or manual vacuum aspiration (MVA) and cannula	153	95.6	208	84.6	X	X	X
Functional suction apparatus for use with catheter and/or manual suction device for fluid extraction	157	98.1	210	85.4	X	X	X
Self-inflating bag and newborn masks (size 0 and size 1) for resuscitation	135	84.4	158	64.2	X	X	X
Resuscitation table/trolley with light source	154	96.3	194/246[1]	79.2			X
Comprehensive EmONC							
Functional sterilizing equipment[3]	139	86.9	--	--	X	X	X
Small and Sick Newborn Care							
Functional incubator	120	75.0	--	--	X		X
Oxygen supply	140	87.5	--	--			X
Pulse oximeter	123	76.9	--	--			X
Amenities – Routine Delivery							
Delivery bed	160	100	244	99.2	X	X	
Private delivery room or visual privacy ensured in delivery area	137	85.6	214	87.0			X
Newborn corner	146	91.3	177	72.0			X
Water outlet on site	156	97.5	208	84.6		X	
Electricity available at all times when facility was open in last 7 days and/or back-up energy source	152	95.0	177	72.0		X	X
Functional toilet available for patient use	160	100	236	95.9		X	
**Staffing & Systems to Support Quality**							
Routine Delivery							
Skilled birth attendant present at facility or on call 24 hours	158	98.8	232	94.3	X	X	X
Ratio of skilled health personnel to delivery volume meets or exceeds desired scenario in FIGO statement[4]	151	94.4	234	95.1			X
Management protocols on selected obstetric topics (FMOH, 2010)	81	50.6	76	30.9	X	X	
Comprehensive EmONC							
Skilled provider available 24h to provide C-section	151	94.4	--	--	X		
At least one staff trained in anesthesia	155	96.9	--	--	X		X

	Hospital N=160		Health center/ clinic N=246		SARA	DHS	WHO standards
	No.	%	No.	%			
Information Systems & Quality Improvement Processes							
Functional mechanism for reporting data on maternal deaths to the Maternal and Perinatal Death Surveillance Response	149/159[1]	93.7	210/233[5]	90.1			X
Conducts maternal death reviews	142/159[1]	89.3	170/231[1,5]	73.6			X
Produces monthly reports for the HMIS and receives feedback that includes recommendations for action	133/159[1]	83.6	223/245[1]	91.0			X
Has a performance monitoring team that meets at least quarterly	142/154[6]	92.2	211/229[1,6]	92.1			X
Referral Systems							
Access to a functional ambulance/car on-site for emergency transportation[7]	146	91.3	143	58.1	X	X	X
Printed referral form observed	127/132[8]	96.2	207/241[8]	85.9			X
Functional system for recording and sharing outcomes of cases referred in and out	134/158[9]	84.8	171/244[9]	70.1			X
**Performance of Signal Functions**							
Routine Newborn Care							
Skin-to-skin care	151	94.4	229/245[1]	93.5		X	
Assist mother to breastfeed	160	100	245	99.6		X	
Basic EmONC							
Provided parenteral anticonvulsants to manage high blood pressure in pregnancy	149	93.1	101	41.1		X	X
Provided parenteral or oral uterotonics to prevent or treat PPH	153	95.6	172	69.9		X	X
Performed manual removal of placenta	152	95.0	181/244[1]	74.2		X	X
Provided instrument/ assisted deliveries	153	95.6	128/244[1]	52.5		X	X
Provided parenteral antibiotics for infections related to pregnancy, abortion, labor or delivery	155	96.9	187/244[1]	76.6		X	X
Performed neonatal resuscitation	156	97.5	178/244[1]	73.0		X	X
Comprehensive EmONC							
Performed blood transfusions for maternity care	125	78.1	--	--		X	X
Performed cesarean deliveries	150	93.8	--	--		X	X
Small and Sick Newborn Care							
Provided antenatal corticosteroids for fetal lung maturation	141	88.1	36/244[1]	14.8			X
Neonatal intensive care provided at facility	143	89.4	--	--			X

[1] Denominator adjusted to exclude responses that are missing, “no response,” or “don’t know”.
[2] Several hospitals (n=8) and health centers/clinics (n=12) do not offer immunization services and were not asked about the availability of BCG vaccine; they are classified as not having vaccines available.
[3] At least one of the following: electric autoclave, non-electric autoclave, or electric dry heat sterilizer. For sterilizing equipment to be considered functional, electric equipment must be observed and assessed as functional. Non-electric equipment must have an observed heat source.

Stierman EK, *et al. BMJ Global Health* 2021; 6:e006698. doi: 10.1136/bmjgh-2021-006698

[4] For hospitals with surgical capacity (i.e., CEmONC), FIGO statement recommends the following desired staffing requirements: at least 5 personnel per shift for ≤1000 deliveries per year; 7 personnel per shift for 1001-2000 deliveries; and 10 personnel per shift for 2001-3000 deliveries, etc. Annual delivery volume is divided by 12 to give monthly estimate. Required staffing numbers are multiplied by two assuming two 12-hour shift pattern. Hospitals must also indicate that they have a skilled provider who can perform C-section present or on call 24h and skilled provider who can administer anesthesia. Skilled health personnel for delivery care include ObGyn, general practitioner (physician), health officer, emergency surgery and obstetrics officer, nurse, and midwife (4).
For health centers with no surgical capacity (i.e., BEmONC), FIGO statement recommends the following desired staffing requirements: at least 3 personnel per shift for ≤1000 deliveries per year; 5 personnel per shift for 1001-2000 deliveries; and 6 personnel per shift for 2001-3000 deliveries. Annual delivery volume is divided by 12 to give monthly estimate. Required staffing numbers are multiplied by two assuming two 12-hour shift pattern. Skilled health personnel for delivery care include ObGyn, general practitioner (physician), health officer, emergency surgery and obstetrics officer, nurse, and midwife (4).
[5] Private clinics (n=13) were not asked about reporting to the Maternal and Perinatal Death Surveillance Response or maternal death reviews. Thus, it is not considered a required input for private clinics to be counted as ready.
[6] Private hospitals (n=6) and private/non-profit clinics/centers (n=16) were not asked about a PMT. Thus, it is not considered a required input for private facilities to be considered ready.
[7] Functional refers to all working status, fuel and driver availability within 15 minutes of need being recognized.
[8] Only facilities that make referrals to other facilities were asked this question; n=33 facilities (28 hospitals and 5 health centers/clinics) do not make referrals. Thus, it is not considered a required input for facilities that do not make referrals.
[9] Only facilities that refer patients to other facilities and/or receive referrals from other facilities were asked about the system for sharing outcomes for cases referred in and out.

Table S7: Differences in childbirth service readiness scores and rankings across indices

	Hospitals (n=160)				Health centers / clinics (n=246)			
	Median	IQR (p25, p75)	Mean	SD	Median	IQR (p25, p75)	Mean	SD
**Difference in scores**								
Simple addition (SARA-WHO standards)	0.00	-0.04, 0.04	0.00	0.06	0.07	0.02, 0.14	0.08	0.09
Weighted addition (SARA-WHO standards)	-0.01	-0.07, 0.03	-0.02	0.07	0.05	-0.03, 0.12	0.05	0.11
PCA (SARA-WHO standards)	-0.02	-0.08, 0.01	-0.04	0.10	0.07	0.01, 0.14	0.08	0.10
Simple addition (DHS-WHO standards)	0.01	-0.02, 0.04	0.01	0.05	0.05	0.00, 0.09	0.05	0.07
Weighted addition (DHS-WHO standards)	0.02	0.00, 0.05	0.03	0.05	0.06	0.03, 0.11	0.07	0.07
PCA (DHS-WHO standards)	0.01	-0.01, 0.04	0.02	0.07	0.00	-0.04, 0.06	0.01	0.09
Simple addition (DHS-SARA)	0.01	-0.02, 0.05	0.01	0.06	-0.03	-0.07, 0.03	-0.03	0.07
Weighted addition (DHS-SARA)	0.03	0.00, 0.09	0.04	0.08	0.02	-0.05, 0.11	0.02	0.11
PCA (DHS-SARA)	0.04	0.01, 0.09	0.06	0.14	-0.06	-0.12, -0.01	-0.07	0.08
**Difference in rankings**								
Simple addition (SARA-WHO standards)	-3	-36, 19.5	-9.6	41.9	-9	-40, 19	-13.9	52.6
Weighted addition (SARA-WHO standards)	-0.5	-36, 23.5	-7.1	44.5	-6.5	-46, 30	-7.9	59.1
PCA (SARA-WHO standards)	1	-34, 25.5	-5.7	44.1	0	-33, 29	-2.8	53.2
Simple addition (DHS-WHO standards)	-6	-27.5, 17	-7.4	38.5	-3.5	-28, 20	-4.4	39.6
Weighted addition (DHS-WHO standards)	-3	-25, 17.5	-4.8	40.9	0	-26, 19	-1.9	35.4
PCA (DHS-WHO standards)	3	-20, 19	-2.4	36.3	1	-23, 23	0.0	42.5
Simple addition (DHS-SARA)	0	-15, 22	2.2	34.6	10	-18, 37	9.5	46.8
Weighted addition (DHS-SARA)	0	-23, 31.5	2.4	49.6	6	-33, 41	6.0	61.5
PCA (DHS-SARA)	2	-18, 19.5	3.3	40.4	0.5	-23, 26	2.7	47.2

Stierman EK, *et al. BMJ Global Health* 2021; 6:e006698. doi: 10.1136/bmjgh-2021-006698

Table S8: Comparison of domain readiness scores (simple addition of items within each domain)

	Hospitals (n=160)					Health centers / clinics (n=246)				
**Domain**	No. items	Cronbach alpha	Median [IQR]	Skewness	Kurtosis	No. items	Cronbach alpha	Median [IQR]	Skewness	Kurtosis
SARA Medicines	8	0.48	1.00 [0.88-1.00]	-1.53	5.38	5	0.43	1.00 [0.80-1.00]	-2.04	9.00
DHS Medicines	6	0.37	0.83 [0.83-1.00]	-1.50	6.25	6	0.45	0.83 [0.67-1.00]	-1.03	4.17
WHO Standards Medicines	17	0.62	0.94 [0.85-1.00]	-2.49	15.16	17	0.76	0.71 [0.59-0.82]	-0.66	3.22
SARA Equipment and Supplies	10	0.38[3]	1.00 [0.90-1.00]	-1.52	5.54	8	0.43	0.88 [0.63-0.88]	-0.63	2.84
DHS Equipment and Supplies	11	0.38[3]	1.00 [0.91-1.00]	-2.11	8.55	10	0.42	0.90 [0.80-0.90]	-0.97	4.16
WHO Standards Equipment, Supplies, Amenities	15	0.50[3]	0.93 [0.87-1.00]	-1.90	8.15	11	0.56	0.82 [0.73-0.91]	-1.00	4.16
DHS General[1]	5	--	1.00 [1.00-1.00]	-2.01	5.91	5	0.25	0.80 [0.60-1.00]	-0.74	3.02
SARA Staffing and Guidelines	4	0.23	0.75 [0.75-1.00]	-1.04	4.75	2	--	0.50 [0.50-1.00]	0.29	2.67
DHS Guidelines[2]	1	--	--	--	--	1	--	--	--	--
WHO Standards Staffing and Systems	10	0.48	1.00 [0.90-1.00]	-1.73	6.39	9	0.59	0.89 [0.78-1.00]	-1.26	4.93
DHS CEmONC Functions	7	0.67	1.00 [0.86-1.00]	-3.25	16.80	5	0.61	0.60 [0.40-0.80]	-0.38	2.15
DHS Newborn Functions	3	--	1.00 [1.00-1.00]	-3.07	10.40	3	0.04	1.00 [0.67-1.00]	-1.13	3.21
WHO Standards Signal Functions	10	0.78	1.00 [0.90-1.00]	-3.33	17.08	7	0.61	0.57 [0.43-0.71]	-0.40	2.34

[1] The DHS general domain includes amenities, transportation, and staff. There is no equivalent in the SARA or WHO standards index. DHS general domain items match items found in the SARA equipment and supplies domain; SARA staffing and guidelines domain; WHO standards equipment, supplies, and amenities domain; and the WHO standards staffing and systems domain.

[2] The DHS guidelines domain is calculated based on one item: Management protocol on selected obstetric topics, 2010 (2). Other items included in the domain as originally conceptualized (e.g., trainings) were not available in the PMA-ET survey.

[3] Delivery kit (sterile clamp and ties) and delivery bed dropped from analysis because constant.

Stierman EK, *et al. BMJ Global Health* 2021; 6:e006698. doi: 10.1136/bmjgh-2021-006698

Table S9a. Spearman's ranked correlation coefficients for hospital domain scores (n=160)

Domain	SARA medicines	DHS medicines	WHO Standards medicines	SARA equipment and supplies	DHS equipment and supplies	WHO standards equipment, supplies, amenities	SARA staffing and guidelines	DHS guidelines	WHO standards staffing and systems	DHS CEmONC functions	DHS newborn functions	WHO standards signal functions
SARA Medicines	1.00											
DHS Medicines	0.26***	1.00										
WHO Standards Medicines	0.29***	0.52***	1.00									
SARA Equipment and Supplies	0.35***	0.13	0.23**	1.00								
DHS Equipment and Supplies	0.31***	0.13	0.22**	0.73***	1.00							
WHO Standards Equipment, Supplies, Amenities	0.34***	0.15	0.26**	0.71***	0.67***	1.00						
SARA Staffing and Guidelines	0.25**	0.11	0.21**	0.35***	0.43***	0.40***	1.00					
DHS Guidelines	0.11	0.03	0.10	0.27***	0.35***	0.30***	0.91***	1.00				
WHO Standards Staffing and Systems	0.28***	0.25**	0.10	0.30***	0.19**	0.23**	0.23**	0.07	1.00			
DHS CEmONC Functions	0.47***	0.03	0.21**	0.27***	0.27***	0.23**	0.18*	0.00	0.30***	1.00		
DHS Newborn Functions	0.11	0.16*	0.08	0.17*	0.18*	0.14	-0.01	-0.11	0.18*	0.15	1.00	
WHO Standards Signal Functions	0.47***	0.00	0.25**	0.28***	0.24**	0.22**	0.21**	0.04	0.29***	0.92***	0.13	1.00

*denotes p<0.05; **denotes p<0.01; ***denotes p<0.001

Table S9b. Spearman's ranked correlation coefficients for health center/clinic domain scores (n=246)

Domain	SARA medicines	DHS medicines	WHO standards medicines	SARA equipment and supplies	DHS equipment and supplies	WHO standards equipment, supplies, amenities	SARA staffing and guidelines	DHS guidelines	WHO standards staffing and systems	DHS CEmONC functions	DHS newborn functions	WHO standards signal functions
SARA Medicines	1.00											
DHS Medicines	0.70***	1.00										
WHO Standards Medicines	0.51***	0.60***	1.00									
SARA Equipment and Supplies	0.25***	0.26***	0.49***	1.00								
DHS Equipment and Supplies	0.25***	0.29***	0.50***	0.86***	1.00							
WHO Standards Equipment, Supplies, Amenities	0.28***	0.31***	0.52***	0.75***	0.81***	1.00						
SARA Staffing and Guidelines	0.28***	0.29***	0.30***	0.30***	0.31***	0.28***	1.00					
DHS Guidelines	0.21**	0.23***	0.24***	0.23***	0.23***	0.23***	0.92***	1.00				
WHO Standards Staffing and Systems	0.30***	0.27***	0.37***	0.43***	0.28***	0.32***	0.29***	0.21**	1.00			
DHS CEmONC Functions	0.31***	0.25***	0.36***	0.24***	0.26***	0.27***	0.25***	0.22***	0.21***	1.00		
DHS Newborn Functions	0.18**	0.09	0.18**	0.18**	0.21***	0.14*	0.07	0.01	0.15*	0.27***	1.00	
WHO Standards Signal Functions	0.29***	0.25***	0.37***	0.28***	0.31***	0.31***	0.23***	0.19**	0.23***	0.95***	0.44***	1.00

*denotes p<0.05; **denotes p<0.01; ***denotes p<0.001

Figure S1: Histograms of childbirth service readiness indices

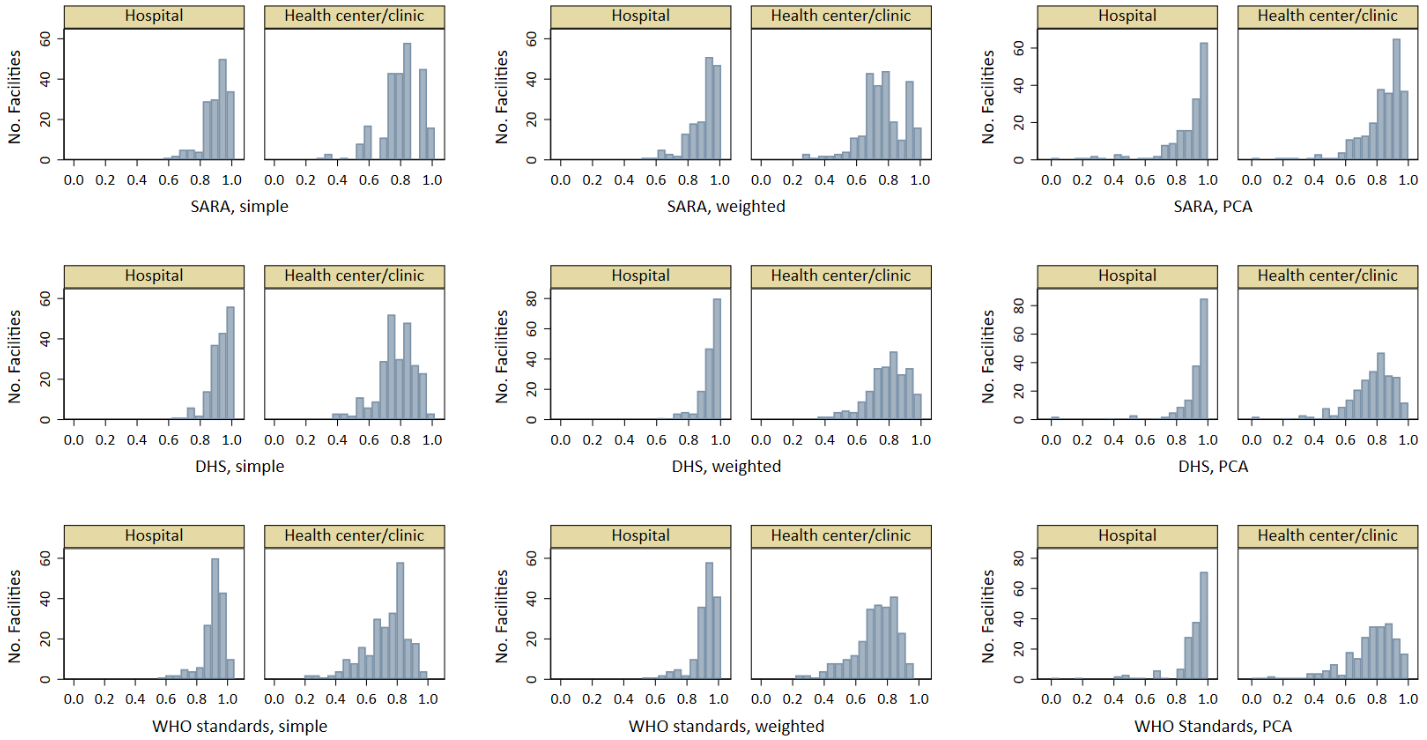

Stierman EK, *et al. BMJ Global Health* 2021; 6:e006698. doi: 10.1136/bmjgh-2021-006698

Figure S2: Boxplots of differences in childbirth service readiness scores and rankings across indices

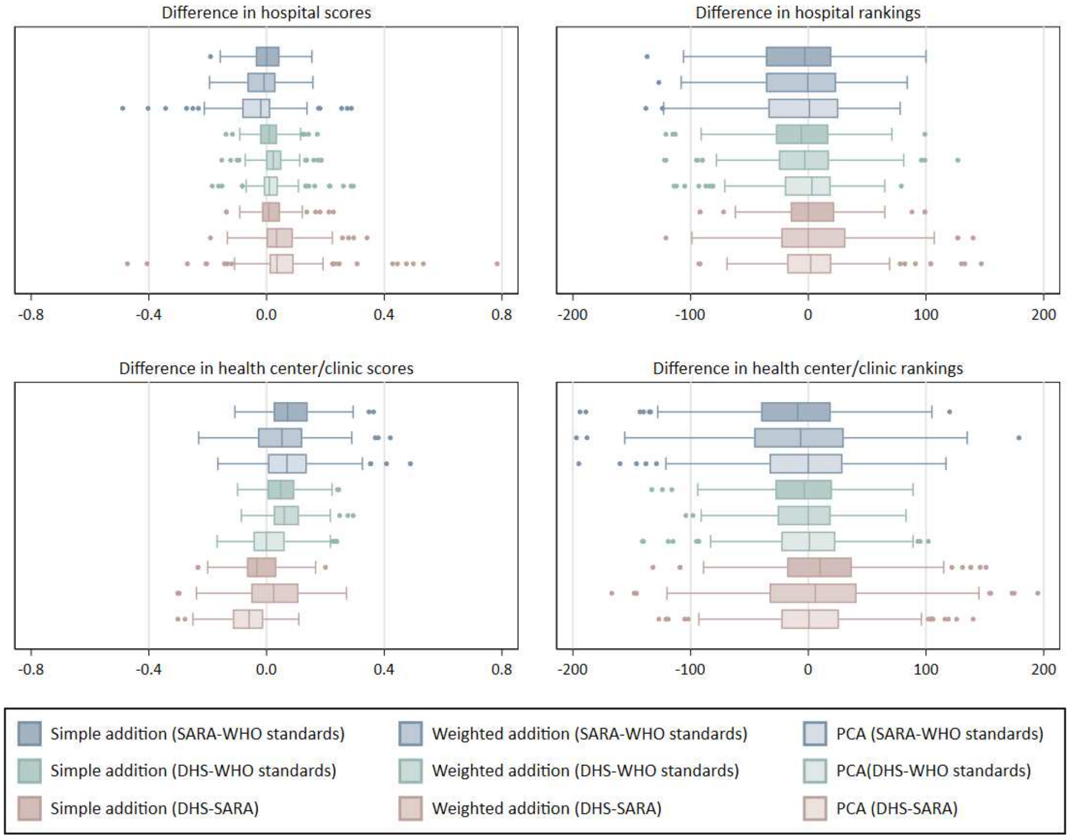

Figure S3A: Correlation between hospital rankings by index

Note: Hospitals are ranked from highest readiness (1) to lowest readiness (160). Ties are awarded 1 + the number of values that are higher.

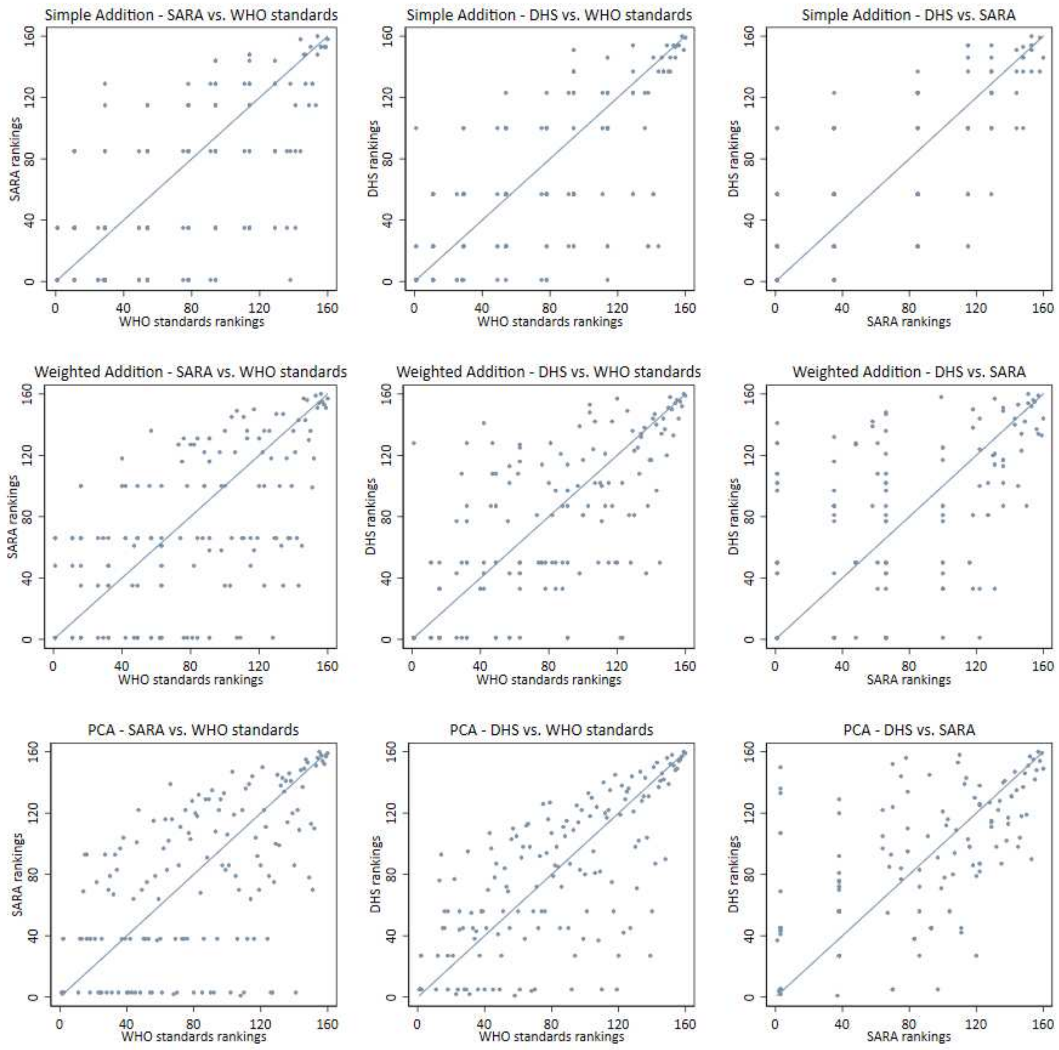

Figure S3B: Correlation between health center/clinic rankings by index

Note: Health centers/clinics are ranked from highest readiness (1) to lowest readiness (246). Ties are awarded 1 + the number of values that are higher.

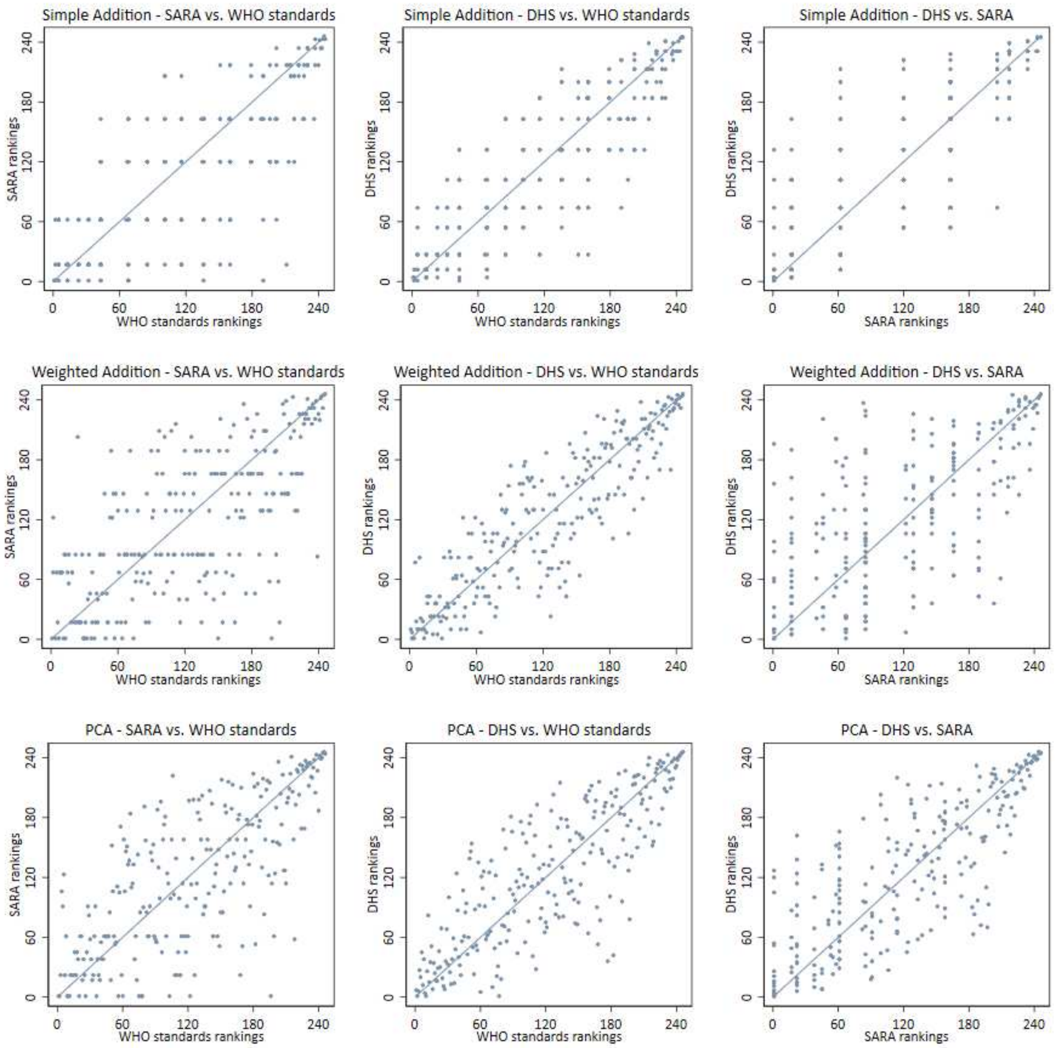

Stierman EK, *et al. BMJ Global Health* 2021; 6:e006698. doi: 10.1136/bmjgh-2021-006698

References

1. World Health Organization. Service Availability and Readiness Assessment (SARA): An annual monitoring system for service delivery reference manual. Geneva, Switzerland: World Health Organization; 2013.
2. Federal Democratic Republic of Ethiopia Ministry of Health. Management protocol on selected obstetric topics. Addis Ababa, Ethiopia: Federal Democratic Republic of Ethiopia Ministry of Health,; 2010.
3. Wang W, Mallick L, Allen C, Pullum T. Effective coverage of facility delivery in Bangladesh, Haiti, Malawi, Nepal, Senegal, and Tanzania. DHS analytical studies no. 65. Rockville, MD, USA; 2018.
4. Stones W, Visser GHA, Theron G for the FIGO Safe Motherhood and Newborn Health Committee. FIGO Statement: Staffing requirements for delivery care, with special reference to low- and middle-income countries. Int J Gynaecol Obstet. 2019;146(1):3-7.
